# Efficacy, mechanisms, and influence factors of small molecule targeted therapy in treating rheumatoid arthritis: a multi-dimensional clinical, immunological, and ultrasonographic study

**DOI:** 10.3389/fphar.2026.1819771

**Published:** 2026-09-28

**Authors:** Zhe Zhou, Yiwen Zhu, Rui Jing

**Affiliations:** 1 Department of Rheumatology and Immunology, Suzhou Ninth People’s Hospital, Suzhou, China; 2 Department of Rheumatology and Immunology, Affiliated Hospital of Nantong University, Nantong, China

**Keywords:** immunological markers, rheumatoid arthritis, rheumatology and immunology, targeted synthetic disease-modifying antirheumatic drugs, ultrasonography

## Abstract

**Background:**

Targeted synthetic disease-modifying antirheumatic drugs (tsDMARDs) show promise for rheumatoid arthritis (RA), but multi-dimensional efficacy evaluation needs further study.

**Objective:**

This study aimed to comprehensively assess tsDMARDs’ efficacy in RA and explore factors linked to treatment response.

**Methods:**

123 consecutive active RA patients treated with tsDMARDs for ≥6 months at Suzhou Ninth People’s Hospital (January 2023–December 2024) were retrospectively analyzed; 120 were included after exclusions. Clinical efficacy was appraised using the Disease Activity Score in 28 joints based on C-reactive protein (DAS28-CRP), Clinical Disease Activity Index (CDAI), American College of Rheumatology 20/50/70 (ACR20/50/70), and the overall effective rate. Immunological parameters included erythrocyte sedimentation rate (ESR), C-reactive protein (CRP), rheumatoid factor (RF), and anti-citrullinated protein antibody (ACPA). Musculoskeletal ultrasound parameters consisted of synovial hypertrophy score assessed by gray-scale ultrasound (GSUS) and synovial vascularity grade assessed by power Doppler ultrasound (PDUS). Correlations between parameters were analyzed, and multivariate logistic regression pinpointed independent risk factors of favorable therapeutic response.

**Results:**

Among 120 patients, patients were stratified into effective (n = 78) and non-effective (n = 42) groups, with comparable baseline characteristics (all *P* > 0.05). After 6 months, the effective group showed significantly greater improvements in DAS28-CRP, CDAI, ESR, CRP, RF, ACPA, GSUS, and PDUS (all *P* < 0.001). Positive correlations were present between immunological/ultrasound parameters and disease activity scores. Multivariate logistic regression identified reduced PDUS signal, lower CRP, and improved DAS28-CRP as independent predictors of favorable response.

**Conclusion:**

tsDMARDs effectively improve joint symptoms, reduce disease activity, and enhance articular function in RA patients, potentially supporting personalized therapeutic strategies.

## 1 Introduction

Rheumatoid arthritis (RA) represents a chronic, progressive autoimmune condition characterized primarily by symmetric polyarticular synovitis. Its pathogenesis is complex, involving immune system dysregulation, abnormal synovial proliferation, and progressive destruction of articular cartilage and bone tissue. Over time, this may trigger joint deformity and functional impairment—outcomes that severely compromise patients’ quality of life while imposing a heavy medical burden on families and society (Di Matteo et al., 2023). Globally, the prevalence of RA ranges from 0.5% to 1.0%, with a rate of approximately 0.42% in China. Driven by an aging population and environmental factors, the RA patient cohort is expanding gradually, making it a priority autoimmune disease for improved diagnosis and treatment strategies (Cush, 2022; Huang et al., 2021).

The therapeutic goals for RA have evolved beyond the traditional focus of relieving joint pain and controlling inflammation. Today, they center on achieving disease remission or low disease activity, preserving joint function, enhancing long-term prognosis, and reducing the incidence of adverse events (Jang et al., 2022). Over the past few decades, RA treatment has undergone remarkable advancements—from symptomatic management with nonsteroidal anti-inflammatory drugs (NSAIDs) and disease-modifying antirheumatic drugs (DMARDs). Conventional synthetic DMARDs (csDMARDs), for short, still serve as the fundamental mainstay in treating RA. Yet, they come with limitations: slow onset of action, inadequate response in some patients, and a risk of hepatorenal damage with long-term use (Venetsanopoulou et al., 2022). The advent of biological agents has offered new options for these non-responsive patients. By specifically targeting key molecules in the inflammatory response, these agents rapidly control inflammation and alleviate joint symptoms. Their clinical utility, however, is constrained by high costs, the need for injectable administration, treatment failure due to anti-drug antibody formation in some patients, and an elevated risk of infections (Wu et al., 2022).

Among targeted synthetic DMARDs (tsDMARDs), Janus kinase inhibitors (JAKi) stand out as the most widely used and extensively studied class of tsDMARDs in clinical practice (Cush, 2021). Compared with biological agents, tsDMARDs boast advantages such as small molecular weight, oral administration, strong penetration, and relatively lower production costs—traits that enhance patient acceptance. They also exhibit precise targeting in their mechanism of action: by regulating immune system signaling pathways with high specificity, they block the initiation and progression of inflammation, inhibit abnormal synovial cell proliferation, and reduce joint tissue damage (Uke et al., 2025; Bugatti et al., 2023). Numerous clinical studies have confirmed that tsDMARDs can significantly alleviate joint symptoms, reduce disease activity, and improve treatment response rates in patients with moderate-to-severe RA (Daikh, 2022; Misra, 2025). That said, most existing research focuses on single-dimensional efficacy assessments—such as changes in clinical symptom scores or individual immunological indicators—lacking comprehensive analysis across clinical, immunological, and imaging dimensions. Additionally, study populations are often limited to specific groups in clinical trials. As a result, the real-world comprehensive efficacy and long-term safety of tsDMARDs still require further verification.

For clinical efficacy evaluation, the Disease Activity Score in 28 joints based on C-reactive protein (DAS28-CRP), Clinical Disease Activity Index (CDAI), and the American College of Rheumatology (ACR) 20/50/70 see broad application in clinical work. These indicators objectively reflect improvements in joint pain, swelling, and other symptoms, as well as changes in disease activity (Liu et al., 2023). Erythrocyte sedimentation rate (ESR) and C-reactive protein (CRP) are classic markers of systemic inflammation, with their levels closely linked to RA inflammatory activity (Kolarz et al., 2021). Rheumatoid factor (RF) and anti-citrullinated protein antibody (ACPA) are characteristic autoantibodies of RA; their titers correlate strongly with disease severity, joint damage progression, and treatment response, serving as important references for evaluating therapeutic effects and prognosis. Musculoskeletal ultrasound—particularly gray-scale ultrasound (GSUS) and power Doppler ultrasound (PDUS)—offers non-invasive, convenient, and repeatable advantages for visualizing pathological changes such as synovial hyperplasia, joint effusion, and cartilage damage. Specifically, GSUS-derived synovial hypertrophy scores quantify the severity of synovial inflammation (Chen et al., 2024), while PDUS-based synovial vascularity grading reflects synovial angiogenesis. Synovial angiogenesis, a key pathological process underlying abnormal synovial proliferation and persistent inflammation in RA (Noto et al., 2025), serves as a valuable imaging indicator for assessing tsDMARD treatment efficacy.

Against this backdrop, this study employed a retrospective analysis approach to enroll patients with active RA receiving tsDMARDs. We comprehensively evaluated the efficacy of tsDMARDs in treating RA across multiple dimensions—including clinical outcomes, immunological indicators, and ultrasound imaging findings. Correlation analysis and multivariate logistic regression were harnessed to explore the intrinsic associations between various indicators and therapeutic effects. The aim was to provide scientific evidence for personalized RA treatment and optimize the clinical application of tsDMARDs.

## 2 Patients and methods

### 2.1 Study design

123 consecutive active RA patients treated with tsDMARDs for ≥6 months at Suzhou Ninth People’s Hospital (January 2023–December 2024) were retrospectively analyzed; 120 were included after exclusions. According to the ACR criteria, the patients were segregated into an effective group (n = 78) and a non-effective group (n = 42) (Figure 1).

**FIGURE 1:**
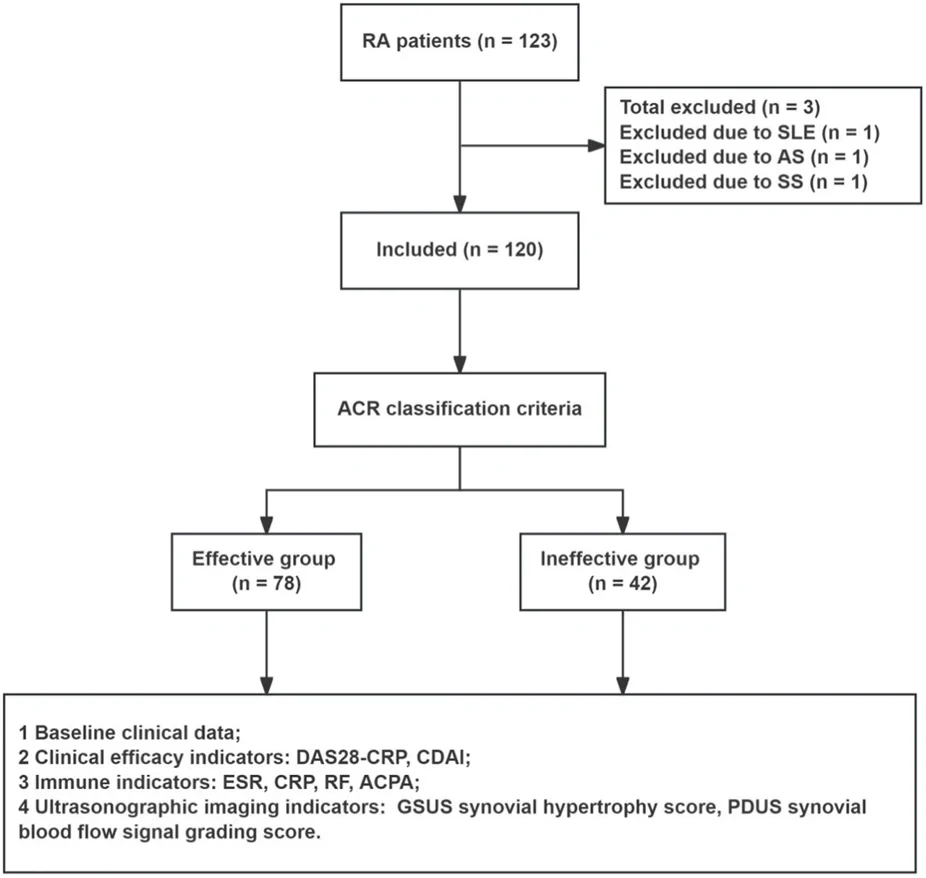
Study flow chart. Note: SLE: Systemic Lupus Erythematosus; AS: Ankylosing Spondylitis; SS: Sjogren’s Syndrome; ACR: American College of Rheumatology; DAS28-CRP: Disease Activity Score 28 - C-reactive protein; CDAI: Clinical Disease Activity Index; ESR: Erythrocyte Sedimentation Rate; CRP: C-reactive protein; RF: Rheumatoid Factor; ACPA: Anti-Citrullinated Protein Antibody; GSUS: Gray - scale Ultrasound; PDUS: Power Doppler Ultrasound.

123 patients were initially screened in this study, after exclusion, 120 cases were included, with 78 cases in effective group and 42 cases in ineffective-group.

### 2.2 Inclusion criteria

(1) Conformed to the diagnostic criteria for RA specified in the 2024 Chinese Guidelines for the Diagnosis and Treatment of Rheumatoid Arthritis (Tian and Zeng, 2024); (2) Presented with active RA, defined as a DAS28-CRP >3.2 or a CDAI >10 points; (3) Received tsDMARDs treatment for ≥6 months with good treatment adherence, including regular medication intake as prescribed without unauthorized drug withdrawal or dose reduction; (4) Had complete clinical data; (5) Were aged 18–75 years.

### 2.3 Exclusion criteria

(1) Complicated with other autoimmune diseases; (2) Had severe joint deformity, a history of joint replacement, or severe bone destruction; (3) Complicated with severe infectious diseases, including active tuberculosis, fungal infections, and viral infections; (5) Received long-term high-dose glucocorticoids or frequent combined immunosuppressant regimens during treatment; (6) Had severe hepatic or renal insufficiency; (7) Had severe hematological abnormalities; (8) Were pregnant or lactating; (9) Complicated with severe underlying diseases (10) Had mental illnesses or cognitive impairments (Wysham et al., 2022).

### 2.4 Treatment regimens

All enrolled patients received treatment in strict conformity with the 2024 Chinese Guidelines for the Diagnosis and Treatment of Rheumatoid Arthritis (Tian and Zeng, 2024). Individualized treatment regimens were formulated based on patients’ baseline disease status, concurrent medications, and individual tolerance, with all patients completing at least six consecutive months of tsDMARDs treatment.

#### 2.4.1 Core drugs

Tofacitinib (HJ20181,078, Pfizer Manufacturing Deutschland GmbH, Germany): The regular dose was 5 mg twice daily. For patients aged >65 years or with mild renal insufficiency, the clinical dose was adjusted to 5 mg once daily to avoid increased infection risk associated with excessive dosage (Harrington et al., 2023).

Baricitinib (H20256,060, Shandong Huabaikasheng Biotechnology Co., Ltd., Shandong, China): The regular dose was 2 mg once daily. For patients with extremely high baseline disease activity, a short-term dose of 4 mg once daily was administered clinically, which was adjusted to 2 mg once daily for maintenance treatment after 4–8 weeks based on disease remission (Harrington et al., 2023).

Upadacitinib (HJ20220,010, AbbVie Deutschland GmbH and Co. KG, Germany): The regular dose was 15 mg once daily (Harrington et al., 2023).

#### 2.4.2 Combined medications

To optimize therapeutic efficacy, reduce adverse reactions caused by excessive single-drug dosage, and avoid drug-drug interactions, individualized combined medication regimens were developed. Based on medical record reviews, the standardized regimens are summarized below, with all combinations complying with clinical practice guidelines.

Some patients received combination therapy with a single csDMARD, and tsDMARDs treatment was initiated only after the combined regimen had been stabilized for ≥4 weeks. Methotrexate (H32026197, Jiangsu Hengrui Medicine Co., Ltd., Jiangsu, China) was dosed at 7.5–15 mg per week. No patients received concurrent treatment with ≥2 csDMARDs to prevent excessive suppression of immune function. None of the enrolled patients were treated with combined biological agents (Smolen et al., 2023).

Patients with significant joint pain received short-term NSAIDs for symptomatic control of pain, with no long-term use records. No patients received long-term high-dose glucocorticoids; for a small number of patients with short-term use, the dosage and duration were fully documented in medical records and included in the analysis of confounding factors (Gaujoux-Viala et al., 2023).

Patients with other underlying diseases (e.g., hypertension, diabetes) continued to take medications for their underlying conditions, with avoidance of drugs that might affect tsDMARDs metabolism. Relevant monitoring records were retained in medical records (Albrecht et al., 2023).

### 2.5 Data extraction

Data were extracted from electronic medical records by two professional researchers independently, with cross-verification. Baseline data were extracted on the day of tsDMARDs initiation and within 1 week before initiation. Outcome data were extracted on the day of completing 6 months of standardized tsDMARDs treatment and within 1 week before and after this time point. Dynamic monitoring data during treatment were also extracted, covering general patient information, efficacy indicators, medication information, and adverse reactions. Strict data quality control was implemented to ensure the accuracy and traceability of the study.

### 2.6 Outcome measures

#### 2.6.1 Clinical disease activity indicators

1. DAS28-CRP score: Completed by professional rheumatologists, DAS28-CRP was derived using a standard formula based on the number of swollen and tender joints among 28 target joints (bilateral proximal interphalangeal joints, metacarpophalangeal joints, wrists, elbows, shoulders, and knees), combined with CRP levels and patient global health assessment (Visual Analogue Scale [VAS] 0–10 points). Elevated scores typically point to a greater degree of disease activity (Binvignat et al., 2024).
2. CDAI score: CDAI was the sum of four indicators: the number of swollen and tender joints among 28 target joints, physician global assessment (VAS 0–10 points), and patient global assessment (VAS 0–10 points). With a total score falling between 0 and 76, elevated scores typically suggest a greater degree of disease activity (Konzett et al., 2024).
3. ACR20/50/70 response rates and overall treatment effective rate: Both are core indicators for evaluating RA treatment efficacy, determined by professional rheumatologists in accordance with criteria established by ACR. For ACR20/50/70 response rates, seven core indicators were compared between baseline and 6 months after treatment: number of swollen joints (28), number of tender joints (28), patient global health assessment (VAS 0–10 points), physician global assessment (VAS 0–10 points), ESR, CRP, and joint function score (assessed by the health assessment questionnaire-disability index). An ACR20 response was defined as an improvement of ≥20% in at least five of the seven indicators, with no indicator worsening by ≥ 20% from baseline. An ACR50 response required an improvement of ≥50% in at least five indicators (with no indicator worsening by ≥ 20%), and an ACR70 response required an improvement of ≥70% in at least five indicators (with no indicator worsening by ≥ 20%). Higher response rates indicate better tsDMARDs treatment efficacy. Overall treatment effectiveness was referred to as achieving ACR20 response at 6 months after treatment, combined with DAS28-CRP ≤3.2 or CDAI ≤10, and no occurrence of severe adverse events (Maeyama et al., 2024).

The composite efficacy criterion used in this study to define a favorable treatment response was constructed based on international regulatory guidance and the treat-to-target paradigm in RA management. Specifically, a patient was classified as a treatment responder if all three of the following conditions were met at 6 months: (1) achievement of an ACR20 response, defined as ≥20% improvement from baseline in both swollen and tender joint counts (28 joints) and in at least three of the five additional core set measures (patient global assessment, physician global assessment, pain VAS, Health Assessment Questionnaire-Disability Index, and acute-phase reactant); (2) attainment of low disease activity (LDA), defined as DAS28-CRP ≤3.2 or CDAI ≤10; and (3) absence of severe adverse events during the treatment period. This composite approach was informed by the European Medicines Agency (EMA) 2017 guideline on RA drug development, which recommends LDA as a primary endpoint at 3–6 months in patients with inadequate response to prior DMARDs, with ACR20/50/70 response rates as secondary endpoints (Aiyegbusi et al., 2022). The combination of ACR20 (which captures the magnitude of improvement from baseline) and LDA status (which captures the absolute disease activity state) addresses two complementary dimensions of treatment response, while the inclusion of safety assessment reflects the fundamental principle of benefit-risk balance in clinical decision-making, particularly given the well-recognized safety profile of JAK inhibitors (Da Silva et al., 2018). This three-element composite criterion aligns with the current treat-to-target strategy in RA, where the ultimate therapeutic goal is remission or at least LDA.

#### 2.6.2 Immunological indicators

1. ESR: Measured using the Westergren method. Five milliliters of venous blood was collected, mixed with sodium citrate anticoagulant, injected into a Westergren sedimentation tube, and measured using an automatic ESR analyzer (ESR-60, Beijing Sckeed Technology Co., Ltd., Beijing, China).
2. CRP: Determined using the immunoturbidimetric method. Fasting venous blood (5 mL) was drawn from each individual, followed by processing to isolate serum samples, and measurements were performed using an automatic biochemical analyzer (ZY-1200, Shanghai Kehua Bio-Engineering Co., Ltd., Shanghai, China).
3. RF and ACPA: Detected using enzyme-linked immunosorbent assay with an automatic electrochemiluminescence immunoanalyzer (Cobas e601, Roche, Switzerland).

#### 2.6.3 Ultrasound imaging indicators

GSUS synovial hypertrophy score and PDUS synovial vascularity grade: Assessed using a color Doppler ultrasound diagnostic instrument (EPIQ7, Philips, Netherlands). Typical affected joints, including bilateral wrists, metacarpophalangeal joints, and proximal interphalangeal joints, were selected for evaluation. Synovial thickness was observed under GSUS and scored on a 0–3 scale (Grade 0: no synovial hypertrophy; Grade 1: mild hypertrophy; Grade 2: moderate hypertrophy; Grade 3: severe hypertrophy) (Sapundzhieva et al., 2022). Synovial blood flow signals were captured under PDUS and graded on a 0–3 scale (Grade 0: no blood flow signals; Grade 1: a small amount of punctate blood flow; Grade 2: a moderate amount of strip-like blood flow; Grade 3: a large amount of reticular blood flow) (Sun et al., 2022). This grading reference aligns with common semi-quantitative evaluation standards for joint synovial lesions in clinical ultrasound practice (Cush, 2022). All musculoskeletal ultrasound examinations were independently performed by two experienced sonographers with more than 5 years of specialized experience in rheumatic musculoskeletal ultrasound. Both examiners were blinded to patients’ clinical profiles, laboratory test results, and treatment group assignments. Prior to the formal study, inter-observer reliability was evaluated using 20 pilot patients. The intraclass correlation coefficient (ICC) for GSUS scores was 0.87 (95% CI 0.80–0.92), and the weighted Kappa value for PDUS scores was 0.84 (95% CI 0.76–0.89). During the formal research phase, ultrasound images of each patient were scored independently by the two sonographers, and the average value was adopted for analysis. In cases where the grading discrepancy reached or exceeded 1 grade, consensus was reached through joint discussion.

### 2.7 Ethical statement

This study was sanctioned by the Medical Ethics Committee of Suzhou Ninth People’s Hospital, and informed consent was waived (approval No.: KYLW 2026-028–01). The study was conducted in strict compliance with the Declaration of Helsinki.

### 2.8 Sample size calculation

As a retrospective study, the sample size was determined by the number of eligible patients with complete clinical data within the predefined study period (January 2023 to December 2024), and a prospective *a priori* sample size calculation could not be performed. The final cohort comprised 120 patients (78 responders and 42 non-responders), yielding an overall event rate of 65.0%. Additionally, the 95% confidence intervals for the odds ratios of the three independent predictors (DAS28-CRP: 0.48–0.72; CRP: 0.88–0.96; PDUS: 0.43–0.68) demonstrated acceptable precision, with widths of 0.24, 0.08, and 0.25, respectively, indicating that the effect estimates were estimated with reasonable stability given the sample size.

### 2.9 Statistical analysis

Logistic regression was used to control for confounding biases in baseline data. Normality testing (Shapiro-Wilk test) and homogeneity of variance testing (Levene test) were first performed on continuous data. Continuous data as specified in normal distribution and homogeneous variance were stated as mean ± standard deviation (mean ± SD), with independent samples t-test for inter-group comparison and paired t-test for intra-group comparison. Continuous data not conforming to normal distribution were stated as median (interquartile range) [M (Q1, Q3)], for comparisons between groups, we applied the Mann-Whitney U test; within-group differences were evaluated *via* the Wilcoxon rank-sum test. Categorical data were quantified as number (percentage) [n (%)], with chi-square test for inter-group comparison. Pearson correlation analysis or Spearman rank was intended to explore the interplay between outcome measures and the efficacy of tsDMARDs in the treatment of RA. Indicators with *P* < 0.05 in univariate analysis were included in the Logistic regression model to identify independent predictive factors for the efficacy of tsDMARDs in RA treatment. To ensure temporal precedence between predictors and outcome definition, and to completely avoid circular reasoning, the multivariate logistic regression model included only baseline (pre-treatment) values of all covariates as independent variables, including baseline DAS28-CRP, baseline CRP, baseline PDUS grade, baseline CDAI, baseline ESR, baseline RF, baseline ACPA, and baseline GSUS. The dependent variable was the composite treatment response grouping (effective vs. non-effective) assessed at 6 months after treatment initiation. Because all predictors were measured before treatment initiation and were independent of the post-treatment outcome definition, this model validly evaluates the predictive value of baseline characteristics for subsequent therapeutic response, rather than providing a circular description of treatment effects. To ensure the robustness of the correlation analysis, 95% confidence intervals for all correlation coefficients will be calculated. Additionally, to detect potential multicollinearity among the independent variables in the logistic regression model, the variance inflation factor (VIF) will be computed for all covariates. A VIF value >10 will be considered indicative of notable multicollinearity, warranting further investigation. All statistical tests were two-tailed, and a *P* value <0.05 was considered statistically significant.

## 3 Results

### 3.1 Baseline data

Between-group comparisons in baseline clinical characteristics are summarized in Table 1. The results revealed no statistically significant differences between the two groups in terms of age, gender distribution, body mass index, comorbidities (hypertension, diabetes), or disease duration (all *P* > 0.05). The two groups had consistent distributions of tsDMARDs types (tofacitinib, baricitinib, upadacitinib), with no patients receiving concurrent treatment with ≥2 tsDMARDs. Additionally, there were no significant inter-group differences in the use of combined single csDMARDs (methotrexate, leflunomide, sulfasalazine), short-term NSAIDs (celecoxib, etoricoxib), or short-term low-dose glucocorticoids (all *P* > 0.05). These findings indicate that the baseline clinical data of the effective group and non-effective group were balanced and comparable, laying a solid foundation for subsequent comparisons of outcome measures.

**TABLE 1:** Baseline clinical data.

Indicators	Effective group (n = 78)	Ineffective group (n = 42)	*P*
Age (years, mean ± SD)	57.28 ± 6.96	58.43 ± 7.12	0.392
Gender (n %)	​	​	0.694
Male	25 (32.05)	12 (28.57)	​
Female	53 (67.95)	30 (71.43)	​
BMI (kg/m^2^, mean ± SD)	24.33 ± 2.42	24.75 ± 2.24	0.352
Hypertension (n %)	18 (23.08)	11 (26.20)	0.704
Diabetes (n %)	10 (12.82)	7 (16.67)	0.565
Course of disease (years, mean ± SD)	8.64 ± 4.30	9.29 ± 4.42	0.436
tsDMARDs (n%)	​	​	0.473
Tofacitinib	31 (39.74)	19 (45.24)	​
Baricitinib	26 (33.33)	14 (33.33)	​
Upadacitinib	21 (26.92)	9 (21.43)	​
Combined with ≥2 tsDMARDs	0 (0.00)	0 (0.00)	​
No combined medication (tsDMARDs monotherapy, n %)	21 (26.92)	15 ()	0.318
Combined with a single csDMARD (n%)	​	​	0.712
Combined with methotrexate	38 (48.72)	16 (35.71)	​
Combined with leflunomide	12 (15.38)	7 (16.67)	​
Combined with sulfasalazine	7 (9.00)	4 (9.52)	​
Combined with short-term NSAIDs (n%)	​	​	0.498
Combined with celecoxib	18 (23.08)	13 (30.95)	​
Combined with etoricoxib	14 (17.95)	8 (19.05)	​
Combined with glucocorticoids (short-term, low-dose, n %)	8 (10.26)	4 (9.52)	0.898

BMI: body mass index; tsDMARDs: targeted synthetic Disease-Modifying Antirheumatic Drugs; csDMARDs: conventional synthetic Disease-Modifying Antirheumatic Drugs; NSAIDs: Non-Steroidal Anti-Inflammatory Drugs.

### 3.2 Clinical efficacy indicators

Before treatment, no statistically intergroup differences were observed in DAS28-CRP or CDAI scores (*P* = 0.264, *P* = 0.699). After 6 months of treatment, both scores decreased significantly relative to baseline in both groups (all *P* < 0.05). Moreover, the effective group exhibited substantially lower DAS28-CRP and CDAI scores than the non-effective group (both *P* < 0.001) (Table 2). These outcomes point to that the tsDMARDs-based treatment regimen exerts a certain inhibitory effect on disease activity in both groups, capable of alleviating disease conditions and reducing disease activity scores to a certain extent.

**TABLE 2:** Comparison of clinical efficacy indicators (mean ± SD).

Indicators	Time	Effective group (n = 78)	Ineffective group (n = 42)	*P*	95% CI of the difference	Cohen’s D
DAS28-CRP (scores)	Before treatment	6.17 ± 0.47	6.30 ± 0.61	0.264	−0.33, 0.09	0.23
	6 months after treatment	2.78 ± 0.57*	5.00 ± 0.61*	<0.001	−2.44, -1.99	3.79
CDAI (scores)	Before treatment	57.35 ± 6.34	57.86 ± 7.84	0.699	−3.12, 2.10	0.07
	6 months after treatment	21.62 ± 5.47*	36.45 ± 6.07*	<0.001	−16.99, -12.68	2.61

*P < 0.05 vs. Before treatment; DAS28-CRP: Disease Activity Score 28 - C-reactive protein; CDAI: clinical disease activity index.

### 3.3 Immunological indicators​

Prior to treatment, no significant changes were found between the effective group and non-effective group in the levels of ESR, CRP, RF, or ACPA (all *P* > 0.05). Following 6 months of treatment, all the aforementioned indicators decreased significantly against baseline in both groups (all *P* < 0.05). Additionally, the effective group showed distinctly lower levels of ESR, CRP, RF, and ACPA than the non-effective group (all *P* < 0.001) (Table 3). The present data demonstrate that the tsDMARDs-centered treatment regimen can effectively suppress inflammatory responses in patients and reduce the levels of RA-specific autoantibodies.

**TABLE 3:** Comparison of immune indicators (mean ± SD).

Indicators	Time	Effective group (n = 78)	Ineffective group (n = 42)	*P*	95% CI of the difference	Cohen’s D
ESR (mm/h)	Before treatment	43.68 ± 5.79	44.05 ± 6.30	0.748	−2.63, 1.89	0.06
	6 months after treatment	22.87 ± 5.80*	34.98 ± 5.72*	<0.001	−14.29, -9.92	2.10
CRP (mg/L)	Before treatment	28.21 ± 5.62	28.48 ± 5.25	0.797	−2.35, 1.81	0.05
	6 months after treatment	12.91 ± 5.74*	21.64 ± 3.64*	<0.001	−10.43, -7.03	1.71
RF (IU/mL)	Before treatment	123.83 ± 42.20	134.48 ± 48.14	0.212	−27.45, 6.17	0.24
	6 months after treatment	64.78 ± 14.36*	110.02 ± 15.25*	<0.001	−50.80, -39.68	3.08
ACPA (U/mL)	Before treatment	109.09 ± 37.88	117.33 ± 41.55	0.274	−23.10, 6.61	0.21
	6 months after treatment	54.78 ± 14.36*	90.02 ± 15.25*	<0.001	−40.80, -29.68	2.40

*P < 0.05 vs. Before treatment; ESR: erythrocyte sedimentation rate; CRP: C-reactive protein; RF: rheumatoid factor; ACPA: Anti-Citrullinated Protein Antibody.

### 3.4 Ultrasound imaging indicators

Before treatment, no statistically meaningful differences were detected between two groups with respect to synovial hypertrophy scores assessed by GSUS or synovial vascularity grades evaluated by PDUS (*P* = 0.566, *P* = 0.749). After 6 months of treatment, both GSUS and PDUS scores fell significantly from baseline in both groups (all *P* < 0.05), with the effective group showing remarkably lower scores for both indicators relative to the non-effective group (both *P* < 0.001). From an imaging perspective, these findings suggest that tsDMARDs can effectively improve local joint structural lesions and reduce inflammatory blood perfusion within the joints (Table 4).

**TABLE 4:** Comparison of ultrasonographic imaging indicators (mean ± SD).

Indicators	Time	Effective group (n = 78)	Ineffective group (n = 42)	*P*	95% CI of the difference	Cohen’s D
GSUS (scores)	Before treatment	3.28 ± 0.82	3.21 ± 0.47	0.566	−0.17, 0.30	0.09
	6 months after treatment	1.68 ± 0.69*	2.86 ± 0.35*	<0.001	−1.37, -0.99	1.97
PDUS (scores)	Before treatment	3.49 ± 0.64	3.52 ± 0.51	0.749	−0.26, 0.19	0.06
	6 months after treatment	1.68 ± 0.69*	2.90 ± 0.30*	<0.001	−1.41, -1.05	2.09

*P < 0.05 vs. Before treatment; GSUS: Gray - scale Ultrasound; PDUS: power doppler ultrasound.

### 3.5 Correlation analysis

Figure 2 presents a Pearson correlation coefficient matrix heatmap for eight clinical or laboratory variables (ESR, CRP, RF, ACPA, DAS28-CRP, CDAI, GSUS, and PDUS). The matrix is symmetric with a diagonal of 1.00, and all 28 off-diagonal variable pairs show positive correlations (blue color scale) with coefficients ranging from 0.55 to 0.98 and no negative correlations. The strongest correlation is between GSUS and PDUS (r = 0.86, 95%CI: 0.803, 0.901, P < 0.001), while RF, ACPA, and DAS28-CRP form a tightly clustered group of very high correlations (r = 0.90–0.94, all P < 0.001). ESR also shows strong positive correlations with CRP (0.88, 95% CI: 0.831, 0.916, P < 0.001), ACPA (0.82, 95% CI: 0.753, 0.875, P < 0.001), CDAI (0.85, 95% CI: 0.792, 0.895,P < 0.001), and RF (0.71, 95% CI: 0.607, 0.792, P < 0.001), as well as moderately strong correlations with DAS28-CRP (0.65, 95% CI: 0.524, 0.742, P < 0.001), PDUS (0.80.95% CI: 0.729, 0.862, P < 0.001), and GSUS (0.81.95% CI: 0.726, 0.860, P < 0.001), suggesting relatively limited concordance between the clinical composite score and ultrasound findings. Overall, traditional serological inflammatory and autoantibody markers show moderate to high positive correlations with composite disease activity scores. Table 5 presents collinearity diagnostics for all baseline variables included in the multivariate logistic regression model, with tolerance and variance inflation factor (VIF) values listed for each indicator. The VIF values of all variables ranged from 4.887 to 9.664, among which CDAI showed the highest VIF of 9.664 and PDUS exhibited the lowest VIF of 4.887; correspondingly, tolerance values varied between 0.103 and 0.205. None of the predictors had a VIF exceeding the commonly adopted critical threshold of 10, indicating that severe multicollinearity was absent among these baseline clinical, serological and ultrasonographic parameters.

**FIGURE 2:**
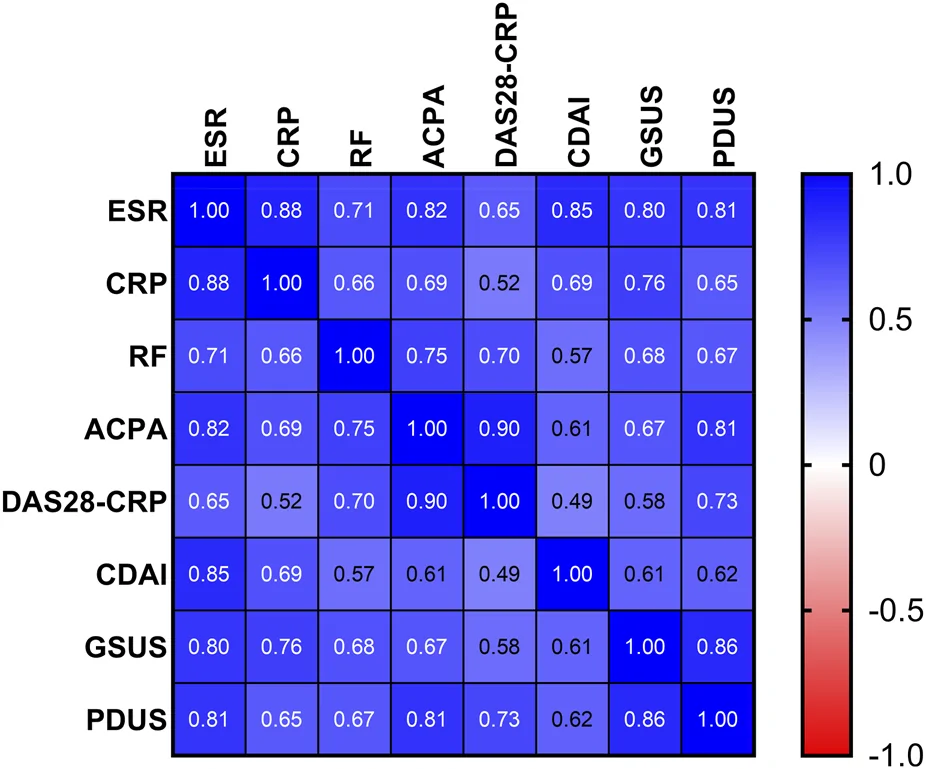
Correlation analysis between various indicators. Note: Rows and columns represent eight evaluation indicators: ESR, CRP, RF, ACPA, DAS28-CRP, CDAI, GSUS and PDUS. Values within each cell denote pairwise Spearman correlation coefficients (r). The color bar on the right visualizes the magnitude of correlation: dark blue indicates strong positive correlation (r = 1.0), fading blue represents weaker positive correlation, white corresponds to zero correlation, and red denotes negative correlation. All pairwise correlations in this cohort were moderate to strong positive associations (r range: 0.49–0.90).

**TABLE 5:** Collinearity diagnostics.

Indicators	Tolerance	VIF
DAS28-CRP	0.128	7.820
CDAI	0.103	9.664
ESR	0.137	7.275
CRP	0.158	6.348
RF	0.118	8.476
ACPA	0.107	9.328
GSUS	0.190	5.271
PDUS	0.205	4.887

VIF: variance inflation factor.

### 3.6 Independent predictive factors for treatment efficacy

Indicators with P < 0.05 selected for the logistic regression analysis to identify independent factors influencing treatment efficacy. In this analysis, all covariates were entered as baseline (pre-treatment) values, while the dependent variable was the 6-month treatment response grouping. The results showed that baseline DAS28-CRP, baseline CRP, and baseline PDUS were independent predictors of treatment outcome (all P < 0.001), with negative associations observed for all three indicators. Specifically, the B value for baseline DAS28-CRP was −0.528 (OR = 0.59, 95% CI: 0.48–0.72); for baseline CRP, the B value was −0.089 (OR = 0.92, 95% CI: 0.88–0.96); and for baseline PDUS, the B value was −0.615 (OR = 0.54, 95% CI: 0.43–0.68). These findings indicate that lower baseline DAS28-CRP scores, baseline CRP levels, and baseline PDUS grades are associated with better treatment responses. In contrast, baseline CDAI, ESR, RF, ACPA, and GSUS had no significant impact on treatment efficacy (all P > 0.05). Although GSUS approached the threshold for statistical significance (*P* = 0.072), it did not meet the criteria for being classified as an independent predictive factor (Table 6).

**TABLE 6:** Analysis of independent risk factors.

Indicators	*B*	*P*	OR	95% for OR
DAS28-CRP (scores)	−0.528	<0.001	0.59	0.48, 0.72
CDAI (scores)	−0.102	0.215	0.90	0.79, 1.04
ESR (mm/h)	−0.015	0.187	0.99	0.97, 1.00
CRP (mg/L)	−0.089	<0.001	0.92	0.88, 0.96
RF (IU/mL)	−0.002	0.356	0.99	0.99, 1.00
ACPA (U/mL)	−0.001	0.428	0.99	0.99, 1.00
GSUS (scores)	−0.165	0.072	0.85	0.72, 1.00
PDUS (scores)	−0.615	<0.001	0.54	0.43, 0.68

## 4 Discussion

This retrospective study analyzed 120 patients with RA who received tsDMARDs treatment for at least 6 months, systematically evaluating the therapeutic efficacy of tsDMARDs and identifying influencing factors from three dimensions: clinical manifestations, immunology, and ultrasound imaging. After 6 months of treatment, the effective group showed significantly greater improvements in DAS28-CRP and CDAI scores than the non-effective group, indicating that tsDMARDs are promising for reducing disease activity and alleviating joint discomfort. For immunological indicators, the effective group exhibited significantly larger reductions in ESR, CRP, RF, and ACPA levels compared with the non-effective group, suggesting that tsDMARDs can effectively suppress abnormal immune responses and reduce inflammatory damage in RA patients. Ultrasound imaging results revealed that the effective group had more pronounced decreases in synovial hypertrophy scores (assessed by GSUS) and synovial vascularity grades (evaluated by PDUS), indicating that tsDMARDs may improve synovial inflammation, reduce synovial blood supply, and delay the progression of joint structural damage. Correlation analysis showed strong positive correlations (r ≥ 0.70) between all immunological indicators, ultrasound indices, and disease activity scores, implying that immune dysregulation and the severity of synovial inflammation may be closely associated with RA disease activity. Multivariate logistic regression analysis indicated that lower baseline PDUS blood flow signals, decreased baseline CRP levels, and lower baseline DAS28-CRP scores may be independent predictive factors for the efficacy of tsDMARDs, providing quantitative references for clinically predicting treatment outcomes and optimizing therapeutic regimens.

tsDMARDs (such as JAK inhibitors) can precisely block key molecules in the pathogenesis of RA, potentially inhibiting inflammatory responses and joint damage at the source (Bernabela and Bermas, 2024). The core pathogenesis of RA may be related to abnormal proliferation of synovial cells, infiltration of immune cells, and massive release of inflammatory factors, forming a vicious cycle of inflammation, synovial hyperplasia, and joint destruction. JAK inhibitors can inhibit the transmission of pro-inflammatory factors, reduce the activation and infiltration of immune cells, thereby suppressing synovial hyperplasia and alleviating joint inflammation. This is consistent with the significant improvements in immunological indicators and ultrasound synovial indices observed in the effective group of this study (Kou et al., 2023). In this study, CRP, PDUS blood flow signals, and DAS28-CRP scores emerged as potential factors related to treatment efficacy, with underlying pathophysiological rationales. As an acute-phase reactive protein, CRP levels can directly reflect the intensity of systemic inflammatory responses. JAK inhibitors may reduce CRP synthesis by inhibiting inflammatory pathways, and a significant decrease in CRP may indicate good inflammatory control, thereby promoting improvements in clinical symptoms (Cao et al., 2024; Pandurangan, 2025). PDUS-assessed synovial blood flow signals are directly associated with the activity of synovial inflammation; reduced blood flow signals may imply decreased synovial angiogenesis and inflammatory cell infiltration, serving as an intuitive imaging marker for the remission of joint inflammation, which may be synchronized with clinical efficacy (Sapundzhieva et al., 2025). The DAS28-CRP score comprehensively reflects disease activity; its degree of improvement may reflect the overall therapeutic effect on the disease and serve as a reference indicator for predicting treatment outcomes. Additionally, the decrease in titers of RF and ACPA—characteristic autoantibodies of RA—was associated with treatment efficacy, suggesting that tsDMARDs can not only control acute inflammation but also regulate abnormal autoimmune responses in the body (Li et al., 2021).

A double-blind phase III study involving Asian populations (Yang et al., 2024) enrolled 385 R A patients with inadequate response to methotrexate, showing that the tsDMARD pixicitinib significantly improved clinical symptoms, with a 6-month ACR20 response rate of nearly 78% and an overall effective rate of approximately 67%. These figures are consistent with the 75.83% ACR20 response rate and 65.00% overall effective rate in this study, indicating that tsDMARDs can maintain good clinical efficacy in real-world settings, with stable targeted mechanisms of action. A retrospective study (Ekin et al., 2024) reported that treatment of RA with the JAK inhibitor tofacitinib led to significant reductions in disease activity indicators at 3 and 6 months after initiation, with sustained significant effects—results consistent with those of the present study. In a multicenter propensity score-matched study (Maeyama et al., 2024), no significant differences were apparent across the normal/mild renal impairment group and the moderate renal impairment group in terms of tender/swollen joint counts, changes in assessor/patient global assessments, rates of achieving low disease activity, or remission/retention rates of clinical/simplified disease activity indices. However, this study focused specifically on RA patients with renal impairment and only included the JAK inhibitor baricitinib, leading to differences from the present study.

The innovations of this study are reflected in three aspects. First, while multidimensional assessments combining clinical, immunological, and ultrasound parameters have been reported in RA research, most existing studies have focused on traditional csDMARDs or biological agents in Western populations. In contrast, this study specifically targeted JAK inhibitors in a Chinese real-world cohort, addressing the paucity of multidimensional predictive evidence for this drug class in East Asian populations. By incorporating clinical efficacy indicators (DAS28-CRP, CDAI), immunological markers (ESR, CRP, RF, ACPA), and ultrasound indices (GSUS and PDUS) into a single evaluation framework, we provide a comprehensive efficacy profile for tsDMARDs in a previously understudied real-world setting. Second, unlike prior studies that typically assessed the predictive value of a single indicator category in isolation, this study systematically compared the independent contributions of all three dimensions by simultaneously entering eight baseline indicators into a single multivariate logistic regression model. This analytical approach enabled us to identify that baseline PDUS—rather than GSUS or traditional serological markers (ESR, CRP, RF, ACPA)—emerged as the dominant independent predictor of favorable treatment response, a finding that would have been obscured in single-modality analyses. This comparative insight offers clinicians a prioritized, quantifiable reference for early identification of patients likely to benefit from tsDMARDs. Third, our findings provide independent, real-world validation of the recently debated question regarding whether baseline ultrasound-detected synovitis predicts response to targeted therapy. While a 2025 study reported that baseline PDUS did not predict treatment response, our results demonstrate that reduced baseline PDUS signal is an independent predictor of favorable efficacy in JAK inhibitor-treated RA patients, contributing new evidence to this ongoing controversy. This may help identify high-risk patients with poor treatment response in advance, enabling timely adjustment of therapeutic strategies and reducing the medical burden and adverse reaction risks associated with ineffective treatment.

## 5 Study limitations and future research directions

This study has several methodological limitations that need to be acknowledged. First and foremost, as a single-center retrospective analysis conducted at one institution in eastern China, the enrolled patient population may not be representative of diverse geographic, ethnic, or healthcare settings, which substantially limits the generalizability of our conclusions. The relatively modest sample size (n = 120) restricts the statistical power for subgroup analyses and multivariate adjustments, and the short follow-up duration of only 6 months is insufficient to evaluate long-term efficacy, sustained remission, radiographic progression, or delayed adverse events. Consequently, our findings should be interpreted with caution and require external validation in larger, multi-center, prospective cohorts with extended observation periods before they can be confidently applied to guide clinical practice. Second, the study included multiple tsDMARDs (tofacitinib, baricitinib, and upadacitinib) without conducting stratified or subgroup analyses by drug type. These factors may affect the representativeness of the study conclusions, particularly in terms of comparing efficacy differences, safety profiles, and influencing factors among different JAK inhibitors. Given the heterogeneity in pharmacological properties, target selectivity, and pharmacokinetics across these agents, the absence of drug-specific subgroup analysis limits the granularity of our findings and their applicability to individualized clinical decision-making. Third, potential influencing factors such as genetic polymorphism and lifestyle were not included. These factors may indirectly affect the efficacy of tsDMARDs by influencing immune function or drug metabolism, leading to incomplete conclusions. Fourth, ultrasound examinations have inherent subjectivity, and different examiners may have variations in judging synovial hypertrophy scores and blood flow signal grades. Although efforts were made to standardize the examination process, subjective errors affecting the results cannot be completely avoided. Fifth, this study was not designed to evaluate the safety profile of tsDMARDs. As a retrospective analysis relying on passive medical record documentation, systematic data on infections (including herpes zoster), cardiovascular events, venous thromboembolism, laboratory abnormalities (including dynamic changes in blood counts, liver and renal function, and lipid profiles), treatment discontinuation with specific reasons, and serious adverse events were not available. The 6-month follow-up period is insufficient to capture rare but serious adverse events such as malignancies or thromboembolic events, which typically require longer exposure and larger sample sizes for reliable assessment. Moreover, with only two laboratory measurement timepoints (baseline and 6 months), transient or subclinical laboratory abnormalities could not be reliably captured. Therefore, our findings should be interpreted exclusively in the context of short-to-medium term efficacy, and no conclusions regarding the comprehensive safety of tsDMARDs can be drawn from this study.

Future research should expand the sample size, conduct multi-center prospective studies, and perform stratified analyses by tsDMARD type to compare efficacy differences, adverse event profiles, and influencing factors among different drugs, thereby enhancing the representativeness and reliability of study conclusions and providing more precise evidence for clinical drug selection. Meanwhile, extending the follow-up period to conduct long-term follow-up assessments will help appraise the long-term efficacy and protective effects of tsDMARDs on joint structural damage, providing references for long-term clinical medication. Subgroup analyses should be carried out to explore differences in tsDMARD efficacy among patients with different disease durations, ages, and disease severities, offering more precise guidance for individualized medication in different populations. Prospective studies with systematic adverse event monitoring and extended follow-up are urgently needed to comprehensively evaluate the benefit-risk profile of tsDMARDs in real-world RA patients.

## 6 Conclusion

tsDMARDs may exert significant therapeutic effects in the treatment of active RA. Reduced PDUS blood flow signals, decreased CRP levels, and improved DAS28-CRP scores may be independent factors associated with the efficacy of tsDMARDs, serving as reference indicators for clinically predicting treatment outcomes and optimizing therapeutic regimens.

## Data Availability

The original contributions presented in the study are included in the article/supplementary material, further inquiries can be directed to the corresponding author.
